# Excessive Extracellular ATP Desensitizes P2Y2 and P2X4 ATP Receptors Provoking Surfactant Impairment Ending in Ventilation-Induced Lung Injury

**DOI:** 10.3390/ijms19041185

**Published:** 2018-04-13

**Authors:** Djo Hasan, Joshua Satalin, Philip van der Zee, Michaela Kollisch-Singule, Paul Blankman, Atsuko Shono, Peter Somhorst, Corstiaan den Uil, Han Meeder, Toru Kotani, Gary F. Nieman

**Affiliations:** 1 Mobile Intensive Care Unit Zuid-West Nederland, 3062 NW Rotterdam, The Netherlands; jmeeder@chello.nl; 2 Department of Surgery, Erasmus MC, Erasmus Universiteit Rotterdam, 3015 CE Rotterdam, The Netherlands; 3 Department of Surgery, Upstate Medical University, Syracuse, NY 13210, USA; satalinj@upstate.edu (J.S.); kolliscm@upstate.edu (M.K.-S.); niemang@upstate.edu (G.F.N.); 4 Adult Intensive Care Unit, Erasmus MC, Erasmus Universiteit Rotterdam, 3015 CE Rotterdam, The Netherlands; p.vanderzee@erasmusmc.nl (P.v.d.Z.); p.somhorst@erasmusmc.nl (P.S.); c.denuil@erasmusmc.nl (C.d.U.); 5 Department of Anesthesiology, Universitair Medisch Centrum Utrecht, 3584 CX Utrecht, The Netherlands; p.blankman@umcutrecht.nl; 6 Department of Anesthesiology, Shimane University, Izumo, Shimane Prefecture 693-0021, Japan; atsukos@med.shimane-u.ac.jp; 7 Department of Cardiology, Erasmus MC, Erasmus Universiteit Rotterdam, 3062 PA Rotterdam, The Netherlands; 8 Department of Anesthesiology and Critical Care Medicine, Showa University, School of Medicine, Tokyo 142-8666, Japan; trkotani@med.showa-u.ac.jp

**Keywords:** extracellular ATP, purinergic signaling, P2X receptors, P2Y receptors, surfactant dysfunction, ventilation-induced lung injury, innate immunity

## Abstract

Stretching the alveolar epithelial type I (AT I) cells controls the intercellular signaling for the exocytosis of surfactant by the AT II cells through the extracellular release of adenosine triphosphate (ATP) (purinergic signaling). Extracellular ATP is cleared by extracellular ATPases, maintaining its homeostasis and enabling the lung to adapt the exocytosis of surfactant to the demand. Vigorous deformation of the AT I cells by high mechanical power ventilation causes a massive release of extracellular ATP beyond the clearance capacity of the extracellular ATPases. When extracellular ATP reaches levels >100 μM, the ATP receptors of the AT II cells become desensitized and surfactant impairment is initiated. The resulting alteration in viscoelastic properties and in alveolar opening and collapse time-constants leads to alveolar collapse and the redistribution of inspired air from the alveoli to the alveolar ducts, which become pathologically dilated. The collapsed alveoli connected to these dilated alveolar ducts are subject to a massive strain, exacerbating the ATP release. After reaching concentrations >300 μM extracellular ATP acts as a danger-associated molecular pattern, causing capillary leakage, alveolar space edema, and further deactivation of surfactant by serum proteins. Decreasing the tidal volume to 6 mL/kg or less at this stage cannot prevent further lung injury.

## 1. Introduction

In 1929, Lohmann discovered and isolated adenosine triphosphate (ATP) from liver and muscles [1,2]. ATP is widely known as an intracellular molecule that is able to transfer energy and is indispensable in living cells [3,4]. Much later, extracellular ATP was identified and appeared to have a different function than the intracellularly located molecule. The intercellular signaling function of extracellular ATP has been described by Felberg and Hebb in 1948 in perfused cervical superior ganglion of the cat [5] and by Holton in 1959 in the sensory nerves of the rabbit ear [6]. In 1972 Burnstock wrote an article on the hypothesis of the purinergic co-transmission in neurons [7]. However, it took more than 20 years before the intracellular energy source ATP was recognized as an extracellular signaling molecule [8]. Now ATP is established as an important element of purinergic signaling in almost all tissues and the immune system [9].

It is well documented that potentially lifesaving mechanical ventilation may ironically damage the lungs and increase mortality risk in patients with acute respiratory distress syndrome (ARDS) by causing ventilation-induced lung injury (VILI) [10,11,12,13] and ARDS remains a serious clinical problem with mortality close to 40% [14]. Gattinoni, et al. (2016) found that it was not just the individual components of the mechanical breath (i.e., tidal volume, respiratory rate, driving pressure and positive end expiratory pressure—PEEP) that cause VILI but rather the mechanical power that the combination of these components generate [11]. Using continuous mandatory ventilation (CMV—volume control ventilation) with a tidal volume of 38 mL/kg (corresponding to a strain of 2.5). Cressoni, et al. (2016) reported that, in piglets, lung injury cannot be provoked at a respiratory rate of ≤9/min corresponding to a mechanical power of <12 J/min [15]. Mechanical power of the ventilator is calculated by a formula. Tidal volume, respiratory system elastance, inspiratory-to-expiratory time ratio, airway resistance, respiratory rate and PEEP are include in the equation of the formula [11]. High power mechanical ventilation is defined as any mechanical breath, which exceeds 12 J/min and corresponds to a mechanical ventilation settings with a tidal volume of >38 mL/kg ideal body weight and a respiratory rate of ≥12/min [15]. Reportedly, intratracheal administration of 400 μL of 5.16 mM ATP in rats leads to alveolar edema [16] and intratracheal instillation of 50 μL of 100 mM ATP or 200 mM uridine triphosphate (UTP) in mice leads to diffuse alveolar damage resembling the effect of high power mechanical ventilation [17]. Recently, we reported that injurious mechanical ventilation results in vigorous cyclic mechanical deformations of the alveolar epithelial cells resulting in massive release of extracellular ATP from the alveolar epithelial type I (AT I) cell [10]. The high levels of extracellular ATP activate a pro-inflammatory immune response of the innate immune system through purinergic signaling [18,19,20] which causes diffuse alveolar damage (DAD) [10,17], the histopathology characteristic of VILI [21].

In this report, we centered on an important component of VILI: the impairment of the pulmonary surfactant function [22,23]. Multiple mechanisms for VILI-induced surfactant impairment in the absence of infection have been reported: (1) increased pulmonary vascular permeability resulting in pulmonary edema with high serum proteins. These serum proteins cause disaggregation and inactivation of surfactant [24], reducing the proportion of functional large aggregates (LAs) significantly in favor of the non-functional small aggregates (SAs) [22,23]; (2) High mechanical power ventilation may enhance the transport of alveolar surfactant into the airways [25,26]. However, these explanations do not account for the mechanism of increased surfactant in the lung lavage in the first two hours during mechanical ventilation with high mechanical power [22,27]. Additionally, the pathogenesis of VILI suggests a mechanism other than capillary leak as the initiating event: High mechanical power ventilation in rats causes surfactant composition changes with surfactant function impairment within one hour and a fall in lung compliance occurs within two hours [22], whereas capillary leak causing overt alveolar edema detected by a computed tomography (CT) scan does not develop for 2–14 h [28] and histological evidence of alveolar edema was not detected at two hours [29].

Therefore, we searched and studied the literature elaborately to find the explanation for the increased surfactant production and the development of surfactant impairment that precede the capillary leak, lung edema and the pro-inflammatory response of the innate immune system.

## 2. Extracellular Release of ATP by AT I and AT II Cells and Clearance of Extracellular ATP

**Figure 1:**
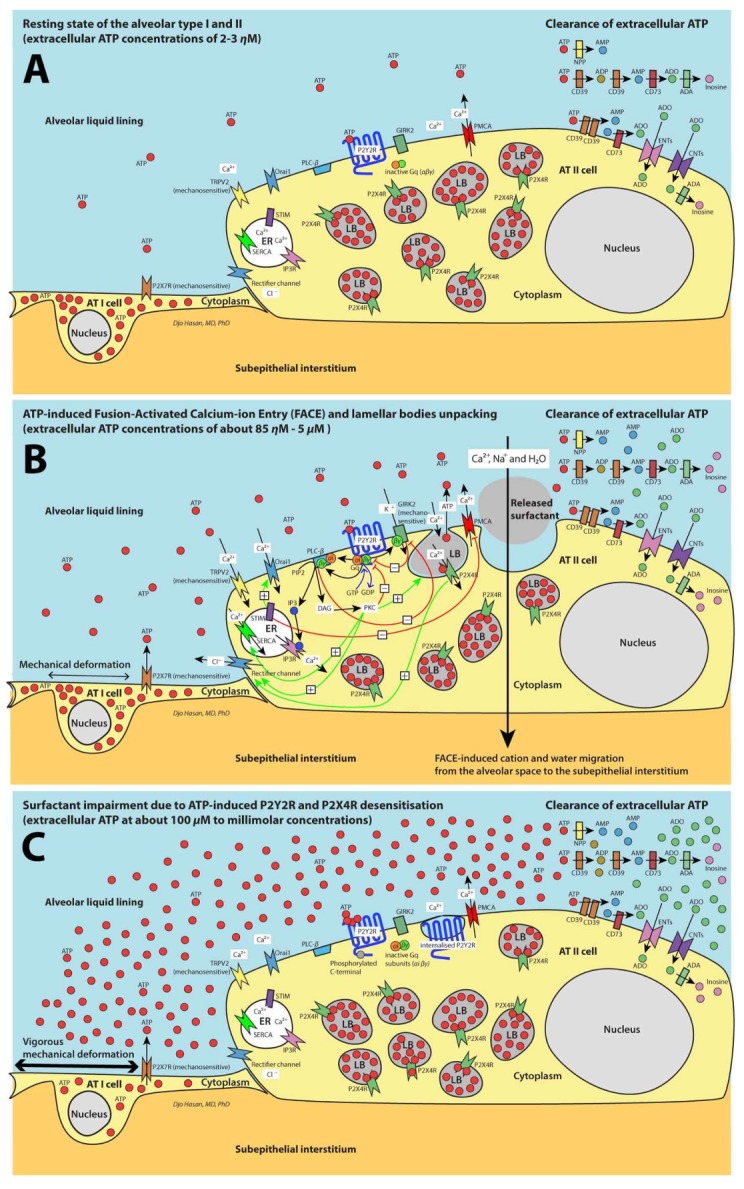
Schematic presentation of the regulation of surfactant exocytosis. For greater clarity, the high cytosolic adenosine triphosphate (ATP) content is omitted in the figure. (**A**) Resting state of the alveolar epithelial type I (AT I) and AT II cells. (**B**) ATP-induced fusion- activated calcium-ion entry resulting in surfactant exocytosis. (**C**) Excessive extracellular ATP concentrations causing the impairment of surfactant exocytosis. See text for explanation. AT I: Alveolar epithelial type I cell; AT II: Alveolar epithelial type II cell; ER: Endoplasmic reticulum; LB: Lamellar body; P2Y2R and P2X4R: ATP receptors; Gq/11: G protein-coupled receptor molecules comprising αi and βγ subunits; PLC-β: Phospholipase C beta; PIP2: Phosphatidylinositol 4,5-bisphosphate; IP3: Inositol triphosphate; IP3R: Inositol triphosphate receptor, a membrane bound glycoprotein complex functioning as a Ca^2+^ channel sensitive to activation by IP3; STIM1: Stromal interaction molecule 1, a calcium sensor; Orai1: Calcium release-activated calcium channel protein 1, a calcium selective ion channel; TRPV2: Transient receptor potential cation channel subfamily V member 2, a non-selective cation channel; Kir3: K^+^ selective inwardly rectifying channel 3 or GIRK2: G protein-coupled inwardly-rectifying K^+^ channel 2; DAG: diacylglycerol; PKC: protein kinase C; CD39: Nucleoside triphosphate diphosphohydrolase 1 (NTPD1); NPP: nucleotide pyrophosphatase/phosphodiesterase; CD73: 5′-nucleotidase (5′-NT); ADA: adenosine deaminase; ENTs: Equilibrative nucleoside transporters 1 and 2; CNTs: Concentrative nucleoside transporters 1 and 2; FACE: fusion-activated Ca^2+^ entry; SERCA: sarcoplasmic/endoplasmic reticulum Ca^2+^ ATPase channel; PMCA: Plasma membrane Ca^2+^ ATPase channel. Figures extensively adapted from Hasan, et al. (2017) [10] (open access) with permission.

In contrast to the relatively high (3 to 10 mM) intracellular concentrations of ATP in epithelial cells [30] the concentration of extracellular ATP in the medium around the 6HBE14o^–^ human bronchial epithelial cells [31] and in resting conditions measured in a cell culture of rat AT II cells [32] is much lower at about 2 nM (Figure 1A). Mechanical deformation (tonic or cyclic stretching) during ventilation of the AT I cells activates the mechanosensitive P2X7 ATP receptors (P2X7Rs) causing a controlled extracellular release of ATP molecules (Figure 1B) [32,33]. In this case the P2X7Rs function as an ATP release channel [34] rather than an intrinsic cation channel or an ATP receptor initiating intracellular signal transduction (P2Y2R and P2X4R in Figure 1B). 

Extracellular ATP molecules are converted by ATP-converting ecto-enzymes or by soluble extracellular enzymes to adenosine (Figure 1A–C) [10,18]. The hydrolyzing enzymes are: Nucleoside triphosphate diphosphohydrolase 1 (NTPD1 or CD39, converts ATP to ADP and ADP to AMP), nucleotide pyrophosphatase/phosphodiesterase (NPP, converts ATP to AMP) and 5′-nucleotidase (5′-NT or CD73, converts AMP to adenosine). Soluble extracellular adenosine deaminase (ADA) converts a proportion of extracellular adenosine to inosine. The remaining adenosine molecule enters the cells via the equilibrative nucleoside transporters (ENT1 and ENT2) and concentrative nucleoside transporters (CNT1 and CNT2). Intracellular adenosine is converted to inosine, hypoxanthine, and AMP by the enzymes ADA, purine nucleoside phosphorylase (PNP) and adenosine kinase (ADK). This process maintains the homeostasis of extracellular ATP in the alveolar walls (Figure 1A–C [10,18].

## 3. Purinergic Signaling Increases the AT II Cytoplasmic Ca^2+^ Levels by the Entry of Extracellular Ca^2+^ and Store-Operated Ca^2+^ Entry (SOCE)

The extent of the extracellular release of ATP molecules by cyclic stretching of the lung is proportional to the strain (equivalent to the tidal volume), frequency and duration of the ventilation [32,33]. When the extracellular ATP concentrations reach the half maximum effective concentration (EC_50_) of 85 to 230 nM in human [35], ATP binds to and activates the P2Y2Rs at the AT II cell membranes (Figure 1B) [36]. This facilitates the coupling of the Gq/11 molecule (comprising αi and βγ subunits) to the G protein-coupled receptor (GPCR) structure of the P2Y2Rs. In the basal state, the heteromeric Gq/11 subunits are indissoluble. After coupling to the GPCR, Gq/11 subunits are activated. The activated αi subunit releases a guanosine 5′-diphosphate (GDP) molecule and binds to a guanosine 5′-triphosphate (GTP) molecule followed by the dissociation of the αi and βγ subunits initiating intracellular signal transduction [37]. The αi and βγ subunits activate phospholipase C beta (PLC-β) to hydrolyze phosphatidylinositol 4,5-bisphosphate (PIP2) resulting in the formation of the second messengers diacylglycerol (DAG) and inositol triphosphate (IP3) [38]. IP3 binds with the IP3 receptors (IP3Rs, a membrane-bound glycoprotein complex functioning as a Ca^2+^ channel sensitive to activation by IP3) causing the release of Ca^2+^ by the endoplasmic reticulum (ER). This process is referred to as store-operated Ca^2+^ entry (SOCE). IP3Rs are important calcium release channels of SOCE [39]. The ryanodine receptor (another important SOCE Ca^2+^ release channel in skeletal muscle, smooth muscle, and cardiac muscle) is not expressed in the lung tissue [40]. SOCE causes Ca^2+^ store depletion that is sensed by the EF-hand and sterile *α* motif (EF-SAM) regions of Stromal interaction molecule 1 (STIM1, a calcium sensor). This information is transferred to activate the plasma membrane STIM1 Orai1-activating region/CRAC-activating domain (SOAR/CAD) regions through the cytoplasmic C-terminus 1 (CC1) regions of the STIM1 molecules located in the cytoplasm. Then the SOAR/CAD regions activate the calcium release-activated calcium channel protein 1 (Orai1) Ca^2+^ channels at the plasma membrane allowing extracellular Ca^2+^ molecules to enter the cytoplasm. STIM1 and Orai1 belong to the calcium release-activated calcium channel (CRAC) family (Figure 1B) [39]. 

**Figure 2:**
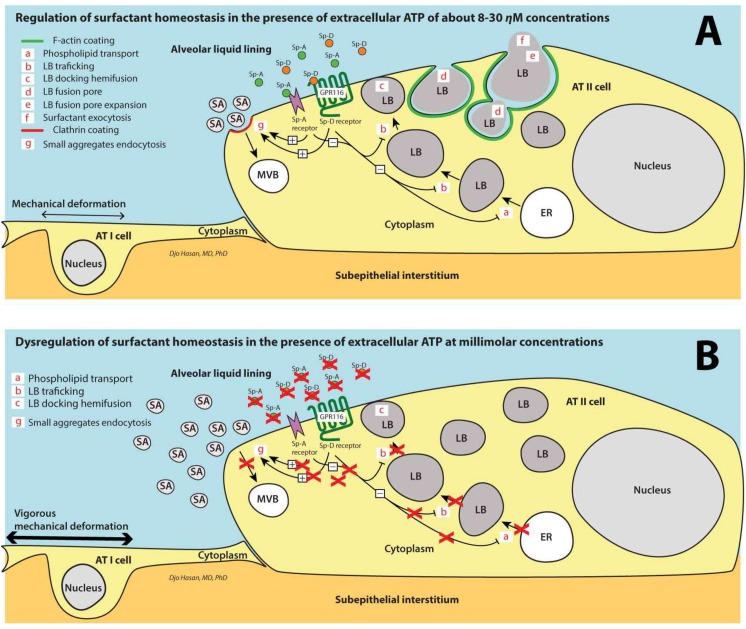
Schematic presentation of the surfactant homeostasis of the alveolar epithelial cells. (**A**) A perivesicular F-actin coating is formed around the fused LBs after the initial LB fusion pore has developed. Several types of fusion pore development are described [57]: (1) 80% of the F-actin-coated fused LBs release surfactant and the LB membrane becomes part of the plasma membrane (kiss-coat-and-release) followed by the disappearance of the F-actin coat; (2) 10% of the F-actin-coated fused LBs discontinued the fusion process and returned inside the cell (kiss-coat-and-run); (3) In the remaining F-actin-coated LBs the fusion process was arrested for a certain time (<20 min) (kiss-coat-and-wait) [57]. The endocytosis of SAs occurs through a clathrin-dependent pathway [59] by the activation of several types of SP-A receptors [59,60,61,62,63] and a SP-D receptor [64]. The SP-D receptor is a GPR116, also known as Ig-Hepta that are highly expressed in the lung [64]. Besides SAs uptake, this process also inhibits the surfactant exocytosis and contributes to the control of extracellular surfactant homeostasis [59,64]. (**B**) The activation of the pro-inflammatory response of the innate immune system through the activation of the P2X7Rs by extracellular ATP at >300 μM concentrations (ATP molecules at these concentrations act as DAMPs) leads to the recruitment and activation of neutrophils. The recruited and activated neutrophils cause the degradation of SP-D and SP-A leading to a deficiency of SP-D and SP-A [65,66] preventing the clathrin-dependent recycling of the majority of SAs and aborting the above-mentioned inhibition of the trafficking, semi-fusion and fusion of the LBs with the cell membrane. AT I: Alveolar epithelial type I cell; AT II: Alveolar epithelial type II cell; ER: Endoplasmic reticulum; LB: Lamellar body; MVB: multivesicular body; SP-A: Surfactant protein A; SP-D: Surfactant protein D; SAs: Surfactant small aggregates; LAs: Surfactant large aggregates; GPR116: G protein-coupled receptor 116; DAMPs: danger-associated molecular patterns.

Inward Ca^2+^ current is also generated though the mechanosensitive transient receptor potential cation channel subfamily V member 2 (TRPV2, a non-selective cation channel) during inspiration [41]. In addition, the Gqβγ subunits of the activated P2Y2Rs simulate (by a direct binding) the K^+^ selective inwardly rectifying channel 3 (Kir3) or G protein-coupled inwardly-rectifying K^+^ channel 2 (GIRK2) expressed on the AT II cell membrane [37]. Kir3 or GIRK2 is a mechanosensitive channel and can also be activated by mechanical deformation of the AT II cells (Figure 1B) [42]. Moreover, activation of the P2Y2Rs and the P2X4Rs induces the volume-regulated anion current channel (VRAC). One of the major components of VRAC is the outwardly rectifying Cl**^–^** channel that is sensitive to protein kinase C (PKC) activation [43,44]. DAG, phosphatidylserine (Ptd-Ser, a component of the AT II cell membrane) and Ca^2+^ are required for the activation of PKC. DAG strikingly increases the affinity of PKC for Ca^2+^ [45]. PKC binds with Ca^2+^ exposing a binding site for Ptd-Ser of the inner part of the cell membrane leading to a redistribution of PKC from the cytosol to the cell membrane [45]. This promotes the trafficking of the lamellar bodies (LBs), docking hemifusion and fusion of the LB membrane with the plasma membrane of the AT II cell (Figure 2A) [46]. After the development of a fusion pore, further pore expansion is accelerated by an additional elevation of cytoplasm Ca^2+^ levels resulting in the exocytosis of surfactant. It was first thought that the additional elevation in Ca^2+^ levels is achieved by extracellular ATP molecules that reach the P2X4Rs located at the LB membrane through the newly formed fusion pore [47,48]. But recently, it appeared that the LBs of rat AT II cells contains a high ATP level of about 1.9 mM at a low pH of 5.5 [49]. ATP is transported from the cytosol to the LBs through the vesicular nucleotide transporter (VNUT) located on the LB membrane [50,51]. P2X4Rs are inwardly rectifying cation (Na^+^ and Ca^2+^) channels located at the membrane of the LBs (Figure 1B) [49]. At pH values lower than 7.4 [49] and at ATP concentrations >100 μM [52] the P2X4Rs are desensitized. Because the fusion pore connects the intravesicular space of the LBs with the extracellular space with a pH value of 7.4 and with low ATP concentrations, the intravesicular pH increases to 7.4 and ATP is released from the LBs to the extracellular space. This causes the intravesicular ATP levels to fall from 1.9 mM to 1–5 μM and within the window of the effective concentrations of the P2X4Rs (as shown in in human embryonic kidney 293—HEK293—cells [52]). This renders the P2X4Rs to become resensitized to ATP stimulation allowing Ca^2+^ ions to enter the cytoplasm (Figure 1B) [52,53].

In addition, IP3 can be transported from AT II to other AT II cells, but direct communication between AT II cells can occur only by bridging the AT I cells that separate the AT II cells by means of tunneling nanotubes (TNTs) [54]. TNTs are long membrane projections with a diameter of 50 to 200 nM and a length of up to ~70 μM (the size of several cells) [55]. TNTs are capable of transporting signals, organelles, and viruses between AT II cells in the presence of connexin gap junction protein isoform 43 (Cx43) [54]. Reportedly, both Cx43 and TNTs are expressed by the AT II cells [56] and intercellular communication through TNTs between alveolar AT II cells that express Cx43 can induce intercellular Ca^2+^ waves by the transmission of IP3 molecules [54]. This process and the activities of outwardly rectifying Cl^–^ channels and G protein-coupled inwardly-rectifying K^+^ channels 2 (GIRK2) reinforce the increase of AT II cytoplasmic Ca^2+^ levels through the paracrine stimulation of the P2Y2Rs by extracellular ATP and the autocrine stimulation of P2X4Rs by vesicular ATP (Figure 1B).

The time required for the LB fusion after the activation of the P2Y2Rs ranges from seconds to several minutes [46]. After the initial fusion pore has developed, a perivesicular F-actin coating is formed around the fused LBs (Figure 2A). This process is Ca^2+^-dependent [57]. Despite the accelerated increase in cytoplasm Ca^2+^ levels, surfactant exocytosis is a relatively slow process (lasting several minutes to hours) [46,58]. The amount of the released surfactant by the AT II cells is proportional to the extracellular ATP levels [32].

## 4. Fusion of Lysosomes and LBs with the Plasma Membrane Plays a Role in the Repair of Damaged Plasma Membrane of the AT I and AT II Cells, Respectively 

Belete, et al. (2011) reported that the repair of damaged rat AT I cell monolayers by applying stretch assay or micropuncture assay is facilitated by the subsequent increase in extracellular ATP concentrations. At extracellular concentrations of about 10 μM ATP activates the P2Y2Rs causing the fusion of the membrane of lysosomes with the plasma membrane releasing the lysosomal-associated membrane protein 1 (LAMP-1) and replacing the damaged plasma membrane [67]. The application of apyrase (CD39) that converts ATP to ADP and AMP and after silencing of the expression of P2Y2Rs the plasma membrane repair rate are reduced significantly [67]. We think that similar repair process of the AT II plasma membrane may occur following the fusion of LBs with the plasma membrane.

## 5. FACE Causes a Trans-Epithelial Transport of Na^+^, Ca^2+^ and Water Molecules

Besides surfactant exocytosis, FACE causes a trans-epithelial transport of Na^+^ and Ca^2+^ molecules from the alveolar space through the P2X4Rs and the cytoplasm of the AT II cells to the sub-epithelial interstitial space. This is followed by a passive water resorption from the alveolar liquid lining to the sub-epithelial interstitial space (Figure 1B). Together with the transepithelial transport of Na^+^ (through the epithelial Na^+^ channel—ENaC) and Cl^–^ (through the cystic fibrosis transmembrane conductance regulator—CFTR) [68] FACE keeps the alveolar liquid lining as thin as 200 nM with a high density of surfactant phospholipid membranes [48]. The thin alveolar liquid lining promotes the contact between the highly organized multilayer surfactant LAs that are stored in the hypophase beneath the surface active monolayer interfacial film and the surfactant monolayer itself. This facilitates the adsorption of surfactant from the multilayer LAs to the surfactant monolayer film [48]. The surface active monolayer interfacial film forms the basis for an optimal diminution of the surface tension of the air-liquid interface in the alveolar space [69]. 

## 6. Surfactant Remodeling in the Alveolar Space

In the alveolar space, surfactant aggregates undergo a remodeling process forming LB-like surfactant compositions and tubular myelin, a lattice-like arrangement of surfactant phospholipid molecules with SP-A and SP-B molecules. These highly organized multilayer surfactant LAs in the extracellular hypophase are thought to be indispensable during the ventilation-induced expansion (inflation) for the adsorption of the phospholipid molecules to the surface active monolayer film at the air-liquid interface (interface film layer) in the alveolar space [69]. SP-B and SP-C are indispensable for the adsorption process [70,71] and this process is followed by the spreading of the surfactant molecules in the interface film layer [69]. Additionally, the transport of oxygen molecules in a water layer containing SP-B and SP-C-mediated densely packed lipid membranes is significantly faster than through a pure water layer or a water layer with pure phospholipid membranes [72]. 

At the beginning of the ventilation-induced compression (deflation) the surfactant molecules in the interface film are not densely packed and still have space to condense causing a rather steep drop in surface tension. Then as deflation progresses the interface monolayer becomes saturated with phospholipid molecules and starts to collapse forming an inward [73,74] or outward [75] buckling of surfactant bilayers. The outwardly buckled surfactant molecules form bilayer disks that rest above the monolayer [75]. These bilayer disks can either be reincorporated into the monolayer [75] or converted to non-functional SAs and lost into the alveolar space and airways [26,69]. Similarly, part of the inwardly buckled surfactant bilayers may be reincorporated into the interface monolayer during the adsorption process or may form new multilayer LAs in the hypophase or may be converted to non-functional SAs [69].

## 7. Surfactant Homeostasis in the Alveolar Space

About 10% of the total surfactant molecules is lost and replenished each hour [69]. A small proportion (7–15%) is cleared through the airways [25,26,69] presumably after the collapse of the interface monolayer through outward buckling [75] and 20% is cleared by macrophages promoted by GM-CSF (granulocyte-macrophage colony stimulating factor) [69]. A very small proportion of surfactant proteins can be detected in the blood [76], but the majority of the “spent” surfactant (about 65%) is taken up by the AT II cells through endocytosis to be recycled [69]. Under basal conditions, SA endocytosis is executed through a clathrin-independent pathway [59]. In contrast, in the presence of secretagogues such as extracellular ATP the uptake of SA is dependent on the clathrin pathway and on both extracellular SP-A and SP-D levels (Figure 2A) [59]. 

Several SP-A-binding proteins at the cell membrane of the AT II cells are reported: (1) SP-A receptors that bind to A2C and A2R monoclonal anti-idiotype antibodies (SPARs) [60]; (2) surfactant-binding protein BP55 [61]; (3) SP-A receptor with a 50-kD protein core that binds SP-A in a calcium-dependent manner not involving the mannose-binding region of SP-A [62]; (4) a 210-kDa SP-R210 [63]; (5) SP-A receptor that is identified as type II transmembrane protein p63 (CKAP4/p63) [59]. Additionally, another receptor that is involved in SA endocytosis is reported: the GPR116 (Figure 2A) [64]. GPR116 is also known as Ig-Hepta which has Ig-like repeats in the N-terminal extracellular domain and is highly expressed in the lung [64]. GPR116 is thought to be an orphan GPCR carrying an agonistic protein sequence (*Stachel* sequence) that functions as a tethered agonist after the removal or a structural change of the N-terminal from the C-terminal fragment of the *Stachel* sequence [77]. Recently, the activation of the GPR116 by synthetic peptides resembling the C-terminal fragment has been reported [77]. In addition, SP-D may function as a ligand activating the GPR116 [64]. Increased SP-D levels in the alveolar liquid lining activate GPR116s. Thus SP-A and SP-D activate the clathrin-dependent ‘spent’ SA uptake and apparently inhibit the exocytosis of surfactant contributing to the control of extracellular surfactant homeostasis in the alveoli (Figure 2A) [59,64].

## 8. Clearance of Ca^2+^ Ions from the Cytoplasm

Clearance of the Ca^2+^ from the AT II cytoplasm occurs by re-entering the endoplasmic reticulum (ER) through sarcoplasmic/endoplasmic reticulum Ca^2+^ ATPase channels (SERCAs) that can transfer Ca^2+^ from the cytoplasm to the ER using energy from ATP hydrolysis [78,79] and/or by leaving the cell through Plasma membrane Ca^2+^ ATPase channels (PMCAs) that are capable of transferring Ca^2+^ from the cytoplasm to the extracellular space (Figure 1B) [80]. Isoforms of both SERCA and PMCA are expressed in the lung [78,80]. Under resting conditions, SERCA is bound to phospholamban (SERCA-PLB complex) and the ATPase activity is inhibited. Activation of SERCA-PLB complex occurs after the cytoplasm levels of Ca^2+^ reach micromolar concentration or after phosphorylation by PKC followed by a partial dissociation of PLB from SERCA [81]. In contrast, PMCA is active under resting conditions and is attenuated by activated STIM1 (Figure 1B) [82]. Restored Ca^2+^ ER levels terminate the stimulation of STIM1 allowing Ca^2+^ ions to be released to the extracellular space. Very high activity of PKC and GIRK2 causes the phosphorylation of the intracellular C-terminal tail of the P2Y2R GPCR molecules causing the ATP receptor to be desensitized (Figure 1B,C) [83,84]. GIRK2 can be inhibited by PLCβ through depletion of PIP2 and activation of PKC (Figure 1B) [42]. These processes control the cytoplasmic Ca^2+^ levels. 

## 9. Ventilation-Induced Extracellular ATP: (1) Initially Increases the Surfactant Release, (2) Halts Surfactant Release and Plasma Membrane Repair at >100 μM Concentrations and (3) Triggers the Pro-Inflammatory Response of the Innate Immunity at >300 μM Concentrations

Mechanical ventilation with high mechanical power (>12 J/min) causes vigorous cyclic deformation of the AT I and AT II cells followed by an increased release of extracellular ATP [10]. This proportionally increases the release of surfactant [32] and explains the increase in LA levels in bronchoalveolar lavage fluids (BALFs) in the first hour during ventilation with high mechanical power [22]. Martinez, et al. (2004) confirmed the increase in surfactant exocytosis and increase in respiratory compliance in the first hour of ventilation with high mechanical power in newborn rats [27]. We postulate that after one hour the purinergic receptors of the surfactant release mechanism become desensitized (Figure 1C). The mechanism for this desensitization could be explained if extracellular levels of ATP reached ≥100 μM: In-vitro exposure of rat glomerular mesangial cells P2Y2Rs to 100 μM ATP during 2 min decreased the sensitivity to stimuli within 1 min. P2Y2Rs reached their maximum desensitization to stimuli within 2 to 4 min. Repetitive stimuli with an interval of 7 min. led to increasingly weaker responses [85]. Desensitization of the P2Y2Rs occurs through two distinct mechanisms: (1) Phosphorylation of the intracellular C-terminal tail of the P2Y2R GPCR by GIRK2 or by PKC. This prevents the coupling of Gqα and GqβY subunits to the P2Y2R GPCR [84]; (2) Internalization of the P2Y2Rs rendering the receptor inaccessible to ATP binding through an unknown pathway [86]. In HEK293 cells desensitization of the P2X4Rs occurs faster within seconds after a stimulus with 100 μM ATP and being maximally desensitized within 30 to 60 s [52]. The mechanisms of desensitization are: (1) Allosteric change of the P2X4R molecules decreasing the Ca^2+^ pore dimensions [53]; (2) Internalization of the P2X4Rs regulated by Rab5 (a small Ras-like GTPase 5) that promote membrane invagination leading to the endocytosis of P2X4Rs through the clathrin pathway [87]. After desensitization, these receptors become unresponsive to ATP stimuli followed by the absence of cytoplasmic Ca^2+^ response to mechanical deformation of the AT I and AT II cell abolishing the surfactant exocytosis by the AT II cells. Therefore, extracellular ATP concentrations >100 μM desensitize the P2Y2Rs at the plasma membrane of AT II cells and prevent the resensitization of the P2X4Rs in the membrane of LBs leading to the impairment of the surfactant release to the extracellular space. Diminishing surfactant exocytosis involves the disappearance of the FACE-induced trans-epithelial transport of Na^+^, Ca^2+^ and water molecules from the alveolar space to the interstitium (Figure 1B,C). Consequently, the thickness of the alveolar liquid lining increases, reducing the density of surfactant phospholipid membranes in the hypophase. This diminishes the contact between both the highly organized multilayer surfactant LAs in the hypophase and the surface active monolayer interfacial film. Obviously, this process contributes to the impairment of surfactant function. We postulate that in addition to a halt in the fusion process of LBs with the plasma membrane of AT II cells, the fusion of lysosomes with the plasma membrane of AT I cells is also inhibited by the desensitization of the P2Y2Rs affecting the capacity of the AT II and AT I cells to repair plasma membrane damage. 

In addition, increasing extracellular ATP levels results in the up-regulation of the ecto-enzymes and soluble extracellular ATP-converting enzymes CD39 and CD73 leading to a significant increase in extracellular adenosine levels [88]. However, the massive release of ATP in high mechanical power ventilation probably exceeds the capacity of these ATP-converting enzymes to convert ATP molecules. The effective extracellular ATP concentrations to activate the P2X7Rs (in JJ4 macrophage cells and in HEK cells expressing P2X7Rs) starts at about 300 to 1000 μM, [89]. In contrast to the P2Y2Rs and P2X4Rs, the P2X7Rs are not subject to desensitization at millimolar or higher extracellular ATP concentrations [89]. P2X7Rs are located at the cell membranes of many immune cells enabling ATP molecules to act as danger associated molecular patterns (DAMPs) activating the pro-inflammatory response of the innate immune system [10,18,19,20]. The recruited and activated neutrophils degrade the SP-D and SP-A molecules through a neutrophil serine protease-dependent cleavage and lead to a deficiency of SP-D and SP-A [65,66]. This prevents the recycling of the majority of SAs (Figure 2B) [59,64]. Under nanomolar concentrations of extracellular ATP deficiency of SP-A and SP-D aborts the inhibition of the surfactant exocytosis (Figure 2A). However, at >100 μM extracellular ATP concentrations the desensitization of the P2Y2Rs and P2X4Rs prevents surfactant release (Figure 2B). The consequences at this stage are that there is a relative increase of non-functional SAs compared to functional LAs and a depletion of LAs in the alveolar space. 

## 10. Surfactant Deactivation Develops Significantly before Alveolar Space Flooding Caused by Increased Capillary Permeability

As mentioned above, it is generally assumed that alveolar exudate of serum proteins explains the deactivation of surfactant function [24] and the development of overt lung edema [15]. However, the group of Lachman (2017) reported that ventilation with high mechanical power for two hours in rats causes a steady increase in the serum C3a levels and a significant increase in lung weight, although the histology of the lung tissue revealed characteristics of diffuse lung injury and a profound interstitial edema they found very little alveolar edema [29]. In addition, Cressoni, et al. (2015) found that alveolar edema as depicted by serial CT scanning as newly developed densities occurs not earlier than 2.1 to 14.7 h [28]. 

We explain this phenomenon by the following consecutive processes: ***First,*** because cytokine levels in mice lung tissue homogenate do not increase earlier than one hour of mechanical ventilation [90] and because the serum complement C3a levels are increased after one hour of high power mechanical ventilation [29], we assume that it takes at more than one hour for the extracellular ATP to reach concentrations >300 μM required to activate the P2X7Rs of the immune cells and initiate the pro-inflammatory response of the innate immune system. ***Second*,** shortly after the activation of the pro-inflammatory response of the innate immunity, complement components are produced by many cells of the immune system. Induction of the activity of complement C5a and C3a in-vitro by moderate concentrations of zymosan (0.01 mg/mL) starts immediately and requires eight hours to reach the maximum level of activation [91]. The small complement fragments C5a and C3a increase vascular permeability in rabbit skin causing capillary leakage of fluid leading to interstitial lung edema [92]. ***Third,*** the tight junctions between alveolar epithelial cells in the lung (consisting of different types of claudins, zonula occludens-1, occludin, etc.) are an important barrier against exudate formation in the alveolar space [93] and claudin-4 and claudin-18 are expressed in the lung tissue [94]. Wray, et al. (2009) reported that the expression of claudin-4 is increased in the course of three hours of mechanical ventilation with a tidal volume of 20 mL/kg [95]. ***Fourth***, the transcription, activation and extracellular release of IL-1β and the IL-1β -dependent production and activation of matrix metalloproteinase 9 (MMP-9) causing the degradation of the tight junctions proteins zonula occludens-1 and occludin [96] requires additional time. This also applies to the P2X7Rs induced-increase in GSK-3β (glycogen synthase kinase 3β) protein levels that reduce the claudin-18 protein levels [94]. Inhibition of the claudin-4 function results in marked increase in alveolar space edema [95].

Therefore, although the interstitial edema caused by capillary leakage occurs immediately after the initialization of the pro-inflammatory response of the innate immune system the breakdown of the tight junctions requires more time. This breakdown of the tight junctions enables the interstitial fluid to reach the alveolar space leading to alveolar edema. This explains the observations that interstitial edema precedes alveolar space flooding by hours [28,29] and provides the evidence that surfactant function impairment that occurs at 2 h after the initiation of high power mechanical ventilation in rats [22] is not caused by the disaggregation of surfactant LAs by the extravasated serum containing serum proteins. This rather early surfactant impairment can be explained by the halted FACE-induced trans-epithelial transport of Na^+^, Ca^2+^ and water molecules as mentioned above. 

## 11. Surfactant Impairment Causes Changes in Alveolar Mechanics Exacerbating the Release of Extracellular ATP

We postulate that the magnitude of the extracellular release of ATP molecules by the mechanical deformation of the alveolar epithelial cells during continuous mandatory ventilation with high tidal volumes and low respiratory rate is such that the capacity of the soluble and ecto-enzymes is sufficient to maintain the extracellular ATP within the concentration range of 85 nM to well below 100 μM. Increasing the respiratory rate to reach a mechanical power of >12 J/min boosts the extracellular ATP release beyond the capacity of the extracellular ATPases (CD39, NPP and CD73) to clear and the extracellular ATP levels gradually increase reaching >100 μM and >300 μM concentrations, resulting in surfactant impairment and VILI, respectively [10,17]. Thus, the healthy lung with intact surfactant function can withstand a strain of 2.5 fairly well [15] as long as the extracellular ATP levels remain below the levels that cause the desensitization of the P2Y2Rs and the PX4Rs [52,85]. 

Using a synchrotron refraction-enhanced computed tomography Sera, et al. (2013) showed that in the healthy murine lung inflation of the lung by increasing the airway pressure from 0 to 8 cm H_2_O changes the alveolar duct diameter and not the alveolar space dimensions. At higher airway pressures, the alveolar ducts diameter remains constant and the alveolar space dimensions increase [97]. Therefore, despite the surfactant-induced decrease in surface tension the alveoli require adequate pressure to increase their diameter. 

In addition to pressure, time is required to inflate the alveoli [98]. This phenomenon is described as the viscoelastic properties of lung tissue by Suki and Bates [99]. The time and the pressure that are required to inflate the alveoli are proportional to the surface tension. The higher the alveolar surface tension the longer the time and the higher the airway pressure that is required for the alveoli to be inflated [98,99]. After surfactant deactivation by saline lavage, a pressure of 40 cm H_2_O over a 2 second period is required to recruit 80% of collapsed alveoli and 40 s to recruit the remaining alveoli [98]. At lower pressures and shorter time intervals, alveoli are not recruited as tidal volume is redistributed towards the alveolar ducts causing a tremendous enlargement of their size [100]. The dramatic increase in the alveolar duct diameter can be explained by the fact that there are 480 million alveoli [101] and 5600 acinar airways including the alveolar ducts [102] with an alveolar:alveolar duct ratio of 2.9 [100]. Redistribution of the tidal volume from the alveoli into the alveolar ducts results in extremely dilated alveolar ducts and pathologic stretching of the alveolar walls of the adjacent alveoli [100].

**Figure 3:**
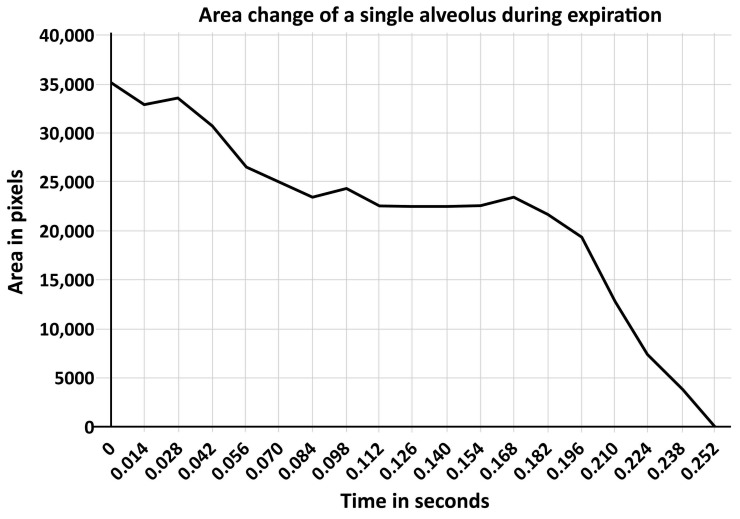
Graphic presentation of the time course of alveolar collapse during the expiration by releasing an airway pressure of 25 cm H_2_O to zero as depicted by *in-vivo* microscopy in rats with surfactant-deactivated lung. The Y-axis represents the alveolar surface areas in pixels and the X-axis is the time. There is a time lag of 0.17 s before alveoli start to collapse after the initiation of the expiratory phase. Furthermore, it takes 0.25 s before the alveoli are fully collapsed. Figure from Satalin, et al. (2016) [103], presented at ‘The Open Forum Sessions’ during the AARC Congress 2016.

**Figure 4:**
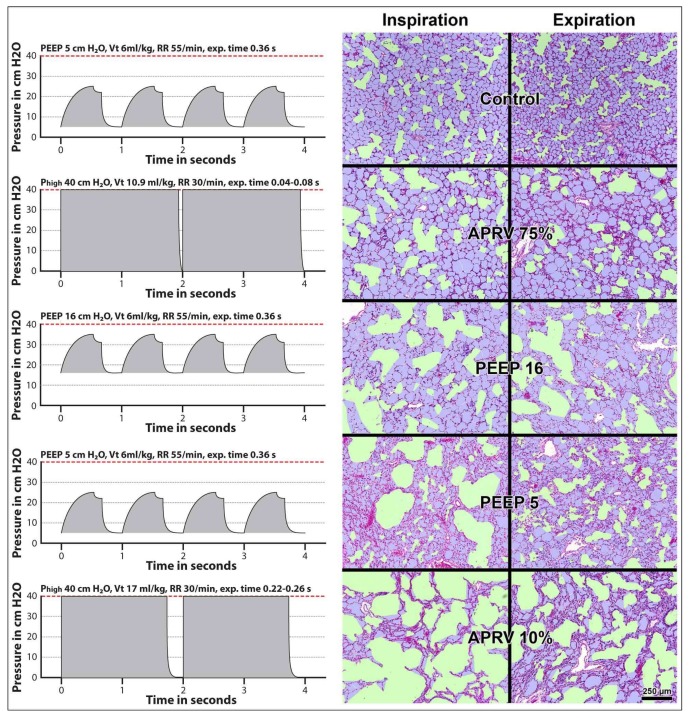
The effect of the ventilator settings on the alveolar mechanics. The left graphics are schematic presentations of the ventilator pressure-time curves belonging to the photomicrographs of the lung presented on the right figure. The lung was fixed at the end-inspiratory pressure (left column of the photomicrographs) and at the end-expiratory pressure (right column of the photomicrographs). The conducting airspaces including the alveolar ducts are colored green, the alveolar spaces are magenta and the alveolar walls are lilac. In APRV75% and APRV10% termination of the expiration is set at an EEF/PEF ratio of 75% and 10%, respectively. In the healthy lung using tissue microscopy after fixing the lung at peak-inspiration and at end-expiration, Kollisch-Singule, et al. (2014) demonstrated that the distribution of tidal volume between the alveoli and the alveolar ducts shows little change during inspiration and expiration (‘control’) [100]. After surfactant deactivation, there is a redistribution of air at the end of expiration from the alveoli towards the alveolar ducts (‘expiration’ and ‘PEEP 5’). During inspiration, the redistribution towards the alveolar ducts markedly increases causing a tremendous deformation of the alveoli adjacent to these alveolar ducts (‘inspiration’ and ‘PEEP 5’). This results in an increased microstrain (defined as the change in length of the alveolar ducts between inspiration and expiration normalized by their original length). Increasing the PEEP level to 16 cm H_2_O decreases the microstrain but not the redistribution of air towards the alveolar ducts (‘Inspiration’, ‘expiration’ and ‘PEEP 16’). The application of APRV10% with a P_high_ of 40 cm H_2_O and expiratory time of 0.22–0.26 s increases the redistribution of air towards the alveolar ducts and the microstrain dramatically (‘Inspiration’, ‘expiration’ and ‘APRV10%’). By applying APRV75% with a P_high_ of 40 cm H_2_O with a shorter expiration time 0f 0.04 to 0.08 s the redistribution of air towards the alveolar ducts and the microstrain much improve but are still not completely restored (‘Inspiration’, ‘expiration’ and ‘APRV75%’) [100]. Thus: in surfactant deactivated lung, a short expiratory time stabilizes the alveoli and a long expiratory time allows alveolar collapse to occur. By setting the timing of the termination of the expiration relative the PEF, the actual expiration time will change proportional to the time-constant of the alveoli. For instance, in slowly deflating alveoli a longer time is required to reach an EEF/PEF ratio of 75% than in fast deflating alveoli. Consequently, the expiration time in a lung with a high compliance is longer than in a lung with a low compliance. Therefore, this mode is now referred to as the ‘time-controlled adaptive ventilation’ (TCAV). APRV: airway pressure release ventilation; EEF: end-expiratory flow; PEF: peak-expiratory flow; P_high_: inspiratory pressure; PEEP: positive end expiratory pressure; Vt: tidal volume; RR: respiratory rate; Exp: Expiratory. Photomicrographs figure from Kollisch-Singule, et al. (2014) [100] with permission.

Moreover, alveoli are not only subject to the viscoelastic properties of the lung during inflation, but also during deflation. Satalin, et al. (2016) reported in a surfactant-deactivated lung that there is a lag time of 0.17 s before alveoli begin to collapse after the termination of the inspiratory phase. Furthermore, it takes 0.25 s before the alveoli fully collapse (Figure 3) [103]. This study demonstrated that the very short expiratory duration using the time-controlled adaptive ventilation (TCAV) protocol (corresponding with the APRV75% group in Figure 4) is critical in normalizing air distribution within the alveoli and alveolar duct in a rat Tween-induced ARDS model and is supported the study by Kollisch-Singule, et al. (2014) and illustrated in Figure 4 [100]. If the expiratory duration is longer than the alveolar collapse-time, these newly recruited alveoli will derecruit with each expiration (Figure 3 and Figure 4); if set shorter than the alveolar collapse-time the alveoli will remain inflated during the brief expiration period (Figure 3 and Figure 4) [100]. Thus, the TCAV protocol stabilizes alveoli by two mechanisms: *pressure* and *time*. Therefore, although ventilation with low mechanical power (<12 J/min) does not cause lung injury in healthy lung [15], ventilation with very low mechanical power corresponding with a tidal volume of 6 mL/kg ideal body weight is injurious for the surfactant-deactivated lung (Figure 3) [100,104]. The efficacy of the TCAV protocol was recently demonstrated in experimental pulmonary and extrapulmonary ARDS [105]. The DAD score (reflecting the extent of pulmonary damage) and the expression of biological markers for lung tissue damage (i.e., amphiregulin, vascular cell adhesion molecule 1—VCAM-1, syndecan 1, metalloproteinase 9—MMP9 and decorin) are significantly higher in volume controlled ventilation (VC) with 8 mL/kg ideal body weight than in TCAV [105]. 

In this perspective, the consequences of mechanical ventilation on the surfactant-deactivated lung are: ***First,*** if the pressure and the duration of the inspiration are inadequate to expand the alveoli tidal volume will be distributed towards the alveolar ducts. This increases the deformation of the alveolar epithelial cells of the adjacent alveoli that are connected to these alveolar ducts tremendously and augments the release of extracellular ATP to a level beyond the capacity of the ATPase enzymes (CD39, NPP and CD 73). ATP will gradually reach >100 μM concentrations causing surfactant impairment and >300 μM concentrations invoking the pro-inflammatory response of the innate immune system injuring the lung tissue. Bellingan, et al. (2014) reported that treatment with interferon-beta-1a (IFN-β-1a) that up-regulates the expression of CD73 reduces the ARDS mortality [106]. ***Second***, even after a successful recruitment maneuver (RM) the newly recruited alveoli will collapse and reopen during every breath if PEEP is not set correctly since the expiratory duration with continuous mandatory ventilation is longer than the alveolar collapse-time. 

The difficulty of opening the lung with a RM and attempting to stabilize it with PEEP was demonstrated in a recent publication by Cavalcanti, et al. (2017) and the “the Alveolar Recruitment for Acute Respiratory Distress Syndrome Trial (ART) Investigators” [107]. They reported a study of patients with moderate to severe ARDS. The patients are randomized into a control and an experimental treatment group. The control arm received ventilation with low tidal volume according to the ARDSNet protocol. The experimental strategy arm received the same low tidal volume protocol as the control group with the addition of neuromuscular blockade and RMs with incremental PEEP up to plateau pressure levels of 50 cm H_2_O followed by a decremental PEEP trial to identify the PEEP level with the highest respiratory compliance. The mean PEEP level in the control group was 12.2 cm H_2_O and 16.8 cm H_2_O in the experimental group. The mean plateau pressure in the experimental group was higher than in the control group but was always below 30 cm H_2_O. There is a slight but statistically significant higher mortality in the experimental group compared to the control group (55.3% vs. 49.3%) [107]. The RMs in the treatment group may open the lung initially, but soon after the termination of the RMs, the newly recruited alveoli recollapse due to an inadequate PEEP level. Therefore, the investigators opened up the lungs during the RM but the level of PEEP failed to keep the lung open thereafter, providing one explanation for the lack of benefit observed in the experimental group. 

## 12. Summary and Conclusions

In the healthy lung, continuous mandatory ventilation with high mechanical power causes an increase in mechanical deformation of the AT I cells followed by an increase in the release of extracellular ATP. This then functions as a signaling molecule for the AT II cells to release surfactant. However, at about 100 μM concentrations, extracellular ATP receptors of the AT II cells become desensitized, surfactant release is halted, and the FACE-induced trans-epithelial transport of Na^+^, Ca^2+^, and water molecules from the alveolar space to the interstitium is diminished, thickening the alveolar liquid lining and impairing the surfactant function. At 300 μM concentrations and above, extracellular ATP initiates the pro-inflammatory response of the innate immune system with immediate increased complement C3 levels causing capillary leakage followed by the disruption of the intercellular junctions of the alveolar epithelial cells, causing overt alveolar space edema. Surfactant disaggregation by serum proteins further deactivates the surfactant function, leading to a significant alteration in the viscoelastic properties of the lung and the redistribution of the tidal volume towards the alveolar ducts. This boosts the extracellular release of ATP by the alveolar epithelial cells and the pro-inflammatory response of the innate immune system. In addition, the initiated pro-inflammatory response of the innate immunity injuring the lung is followed by a reactive adenosynergic immune paralysis of the immune system and fibrosis [10]. Although extracellular ATP levels can be reduced by a treatment with IFN-β-1a, this may increase adenosine levels.

Future research should be directed into blocking high levels of extracellular ATP combined with improved ventilation strategies. Furthermore, new monitoring systems have to be developed to assess markers of the massively increased purinergic signaling in the lung.
